# Whole Genome Sequencing of Familial Non-Medullary Thyroid Cancer Identifies Germline Alterations in MAPK/ERK and PI3K/AKT Signaling Pathways

**DOI:** 10.3390/biom9100605

**Published:** 2019-10-13

**Authors:** Aayushi Srivastava, Abhishek Kumar, Sara Giangiobbe, Elena Bonora, Kari Hemminki, Asta Försti, Obul Reddy Bandapalli

**Affiliations:** 1 Division of Molecular Genetic Epidemiology, German Cancer Research Center (DKFZ), D-69120 Heidelberg, Germany; a.srivastava@dkfz.de (A.S.); abhishek.abhishekkumar@gmail.com (A.K.); sara.giangiobbe@gmail.com (S.G.); k.hemminki@dkfz.de (K.H.); a.foersti@kitz-heidelberg.de (A.F.); 2 Hopp Children’s Cancer Center (KiTZ), D-69120 Heidelberg, Germany; 3 Division of Pediatric Neurooncology, German Cancer Research Center (DKFZ), German Cancer Consortium (DKTK), D-69120 Heidelberg, Germany; 4 Medical Faculty, Heidelberg University, D-69120 Heidelberg, Germany; 5 Institute of Bioinformatics, International Technology Park, Bangalore 560066, India; 6 Manipal Academy of Higher Education (MAHE), Manipal, Karnataka 576104, India; 7 S.Orsola-Malphigi Hospital, Unit of Medical Genetics, 40138 Bologna, Italy; elena.bonora6@unibo.it

**Keywords:** papillary thyroid cancer, germline mutations, whole genome sequencing, predisposition markers, pathway analysis

## Abstract

Evidence of familial inheritance in non-medullary thyroid cancer (NMTC) has accumulated over the last few decades. However, known variants account for a very small percentage of the genetic burden. Here, we focused on the identification of common pathways and networks enriched in NMTC families to better understand its pathogenesis with the final aim of identifying one novel high/moderate-penetrance germline predisposition variant segregating with the disease in each studied family. We performed whole genome sequencing on 23 affected and 3 unaffected family members from five NMTC-prone families and prioritized the identified variants using our Familial Cancer Variant Prioritization Pipeline (FCVPPv2). In total, 31 coding variants and 39 variants located in upstream, downstream, 5′ or 3′ untranslated regions passed FCVPPv2 filtering. Altogether, 210 genes affected by variants that passed the first three steps of the FCVPPv2 were analyzed using Ingenuity Pathway Analysis software. These genes were enriched in tumorigenic signaling pathways mediated by receptor tyrosine kinases and G-protein coupled receptors, implicating a central role of PI3K/AKT and MAPK/ERK signaling in familial NMTC. Our approach can facilitate the identification and functional validation of causal variants in each family as well as the screening and genetic counseling of other individuals at risk of developing NMTC.

## 1. Introduction

Thyroid cancer is the most common endocrine malignancy with an age adjusted incidence of 0.5-20/100,000 persons per year [1]. Significant regional differences exist with Italy being among the countries with the highest incidence rates in the world [1]. An increasing incidence has been observed worldwide during the past decades, which can to a certain extent be related to changes in the availability of medical services and in standard clinical practice. On the other hand, regional differences in incidence as well as changes over time may also be related to lifestyle, nutritional iodine, ionizing radiation and genetic factors [2]. For instance, the high incidence of thyroid cancer in Italy can be attributed to the disruptive and carcinogenic effect of volcanic environments on the endocrine system [3]. The familial relative risk of developing thyroid cancer is estimated to be increased 6.7-fold in a study based on the Swedish Family-Cancer Database, in which 3.4% of all thyroid cancer cases had a concordant family history [4].

Approximately 90–95% of all thyroid cancers are non-medullary thyroid cancers (NMTC) [5] and can be classified into four histological subtypes: papillary, follicular, Hürthle cell and anaplastic thyroid cancer, with papillary thyroid cancer (PTC) being the most common one. Familial NMTC (FNMTC) accounts for only a small percentage of all NMTCs and can be divided into non-syndromic and syndromic forms. In the first, it occurs as the primary feature and in the second, as a minor component of a familial cancer syndrome, such as familial adenomatous polyposis, Gardner’s syndrome, Cowden’s disease, Carney’s complex type 1, Werner’s syndrome, papillary renal neoplasia, and *DICER1* syndrome [6]. Known syndromes explain only a small proportion of all FNMTCs.

Unlike the case of familial medullary thyroid cancer, in which there is extensive evidence linking germline point mutations in the *RET* proto-oncogene to the development of thyroid cancer, the genetic causes for FNMTC remain largely unknown. Over the years, studies seeking genetic factors predisposing to NMTC have been performed using linkage analysis, candidate gene sequencing and recently also whole genome sequencing. These studies have suggested several genes as potential NMTC-predisposing genes, including, *FOXE1*, *SRGAP1*, *TITF-1/NKX2.*1, *SRRM2*, and *HABP2* [7,8,9,10,11]. In addition, an imbalance of the telomere-telomerase complex has been demonstrated in the peripheral blood of familial PTC patients [12]. Nonetheless, NMTC is one of the most heritable cancers wherein first degree relatives of an affected individual have an 8-10-fold increased risk of developing the disease [13]. Therefore, there are many underlying germline mutations that are yet to be discovered.

The identification of such predisposition genes could be of great value in the screening of individuals at risk of developing NMTCs as well as in the development of personalized adjuvant therapies based on the affected pathways. It has been observed that hereditary NMTC is characterized by early onset, a higher degree of aggressiveness and more frequent multifocal disease and recurrence compared with its sporadic counterpart [13]. Thus, medical centers recommend more aggressive treatment of affected family members, reinforcing the importance of identifying such cases.

Here we report the germline genomic landscape of five families with NMTC aggregation consistent with an autosomal dominant pattern of inheritance. The aim of the current study was to use whole genome sequencing (WGS) data to discern pathways affected in the FNMTC families to facilitate the identification of possible disease-causing high/moderate-penetrance germline variants in each family. With our results, we hope to facilitate genetic counseling and targeted therapy in these families and improve screening of other individuals at risk of developing NMTC.

## 2. Materials and Methods

### 2.1. Ethical Approval

Blood samples were collected from the participants with informed consent following ethical guidelines approved by “Comitato Etico Indipendente dell ‘Azienda Ospedaliero-Universitaria di Bologna, Policlinico S. Orsola-Malpighi (Bologna, Italy)” and “comité utiltative de protection des personnes dans la recherche biomédicale, Le centre de ute contre le cancer Léon-Bérard (Lyon, France)”.

### 2.2. NMTC Families

**Figure 1:**
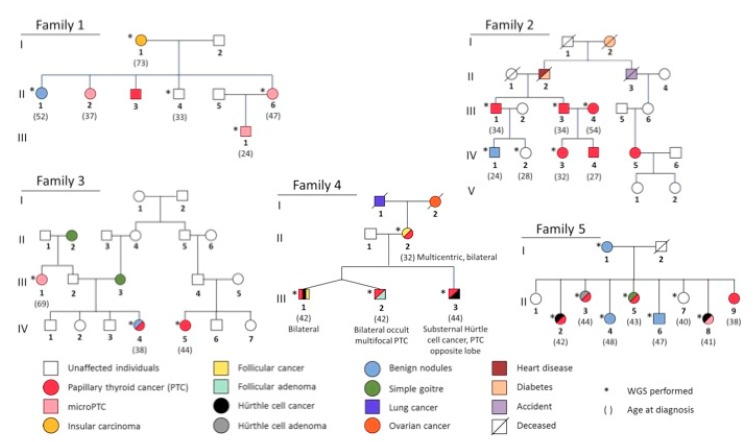
Pedigrees of the five non-medullary thyroid cancer (NMTC)-prone families analyzed in this study.

Five families with NMTC aggregation consistent with an autosomal dominant pattern of inheritance were provided by the S. Orsola-Malpighi Hospital, Unit of Medical Genetics in Bologna, Italy. Samples from a total of 23 affected and 3 unaffected family members from the five families were submitted for WGS. Their respective pedigrees are shown in Figure 1. In family 1, the mother (I-1) was affected by insular carcinoma of the thyroid whereas three of her children and her grandchild were diagnosed with PTC or micro-PTC (II-2, II-3, II-6, III-1) and one child with benign nodules (II-1). Her unaffected son was deemed a reliable control (II-4). WGS (*) was performed on five family members. In family 2, there were six cases (III-1, III-3, III-4, IV-3, IV-4, IV-5), one probable case (IV-1) and one control (IV-2) out of which six underwent WGS. Family 3 consisted of two related cases (IV-4, IV-5) and one unrelated case (III-1) of which all three underwent WGS. Family 4 is characterized by bilateral PTCs concurrent with other subtypes of NMTCs (Hürthle cell cancer, follicular cancer). Four family members were diagnosed with thyroid cancer of which all underwent WGS (II-2, III-1, III-2, III-3). WGS was performed on eight family members of family 5. Five members were affected by PTC, Hürthle cell cancer, micro-PTC or a combination of two of the subtypes (II-2, II-3, II-5, II-8, II-9). Four members were possible carriers either affected by benign nodules or deceased (I-1, II-4, II-6) and two were unaffected (II-1, II-7).

### 2.3. Whole Genome Sequencing and Variant Evaluation

WGS for 23 cases and 3 controls was performed using Illumina-based small read sequencing after DNA was isolated from peripheral blood using the QIAamp ^®^ DNA Mini Kit (Qiagen, Cat No. 51104) according to the manufacturer’s instructions.

### 2.4. Variant Calling Annotation and Filtering

Sequencing data was mapped to a reference human genome (assembly version Hs37d5) using BWA mem (version 0.7.8) and duplicates were removed using biobambam (version 0.0.148). Single nucleotide variants (SNVs) and indels were called from all the samples in a family together using Platypus (version 0.8.1). ANNOVAR, 1000 Genomes, dbSNP and ExAC (Exome Aggregation Consortium) were used in the annotation of variants as explained in detail in our previous paper [14]. Variants to be evaluated further were selected using the following criteria: (i) A quality score greater than 20, and a coverage greater than 5x; (ii) All Platypus filters were met. Variants with a minor allele frequency (MAF) less than 0.1 % in 1000 genome and ExAC-nonTCGA data were selected for further analysis. A pairwise comparison of shared rare variants among the cohort was performed to check for sample swaps and family relatedness.

### 2.5. Variant Filtering following the FCVPPv2

**Figure 2:**
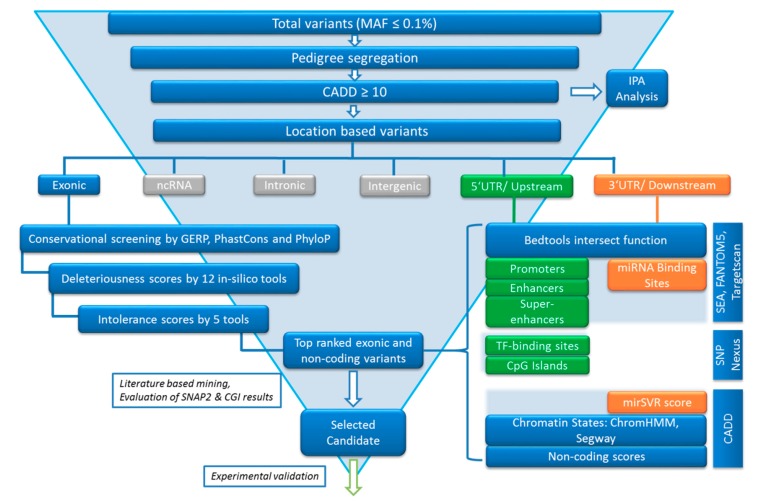
Summary of the familial cancer variant prioritization pipeline version 2 (FCVPPv2).

Variant evaluation was performed using the criteria of our in-house developed Familial Cancer Variant Prioritization Pipeline v2 (FCVPPv2) [14]. This process is summarized in Figure 2 and explained in the following text.

#### 2.5.1. Segregation in Pedigrees

The variants were filtered based on pedigree data considering family members diagnosed with NMTC or micro-PTC as cases, benign nodules or goiter as potential variant carriers and unaffected members as controls. The probability of an individual being a Mendelian case or true control was considered. The general rule was that variants had to be present in all cases and absent from all controls.

#### 2.5.2. Variant Ranking Using In Silico Tools

After filtering variants based on pedigree segregation, the CADD tool v1.3 [15] was applied. Variants with a scaled PHRED-like CADD score greater than 10, which accounts for the top 10% of probable deleterious variants in the human genome, were prioritized. Variants were then selected according to their conservation scores. High evolutionary conservation suggests functional importance of a position. Genomic Evolutionary Rate Profiling (GERP), PhastCons and PhyloP were used to assess conservation of the variant position, whereby GERP scores >2.0, PhastCons scores >0.3 and PhyloP scores >3.0 indicate a high level of conservation and are therefore used as thresholds in the selection of potentially causative variants. After that, all missense variants were assessed for deleteriousness using the following tools: SIFT, PolyPhen V2-HDIV, PolyPhen V2-HVAR, LRT, MutationTaster, Mutation Assessor, FATHMM, MetaSVM, MetLR, PROVEAN, VEST3 and RI using dbNSFP [16]. Variants predicted to be deleterious by at least 60% of these tools were shortlisted for further analysis. Lastly, intolerance scores were considered. These were merely used to rank the variants and not as cutoffs for selection. The ranking of variants according to the intolerance scores of the corresponding genes relies on the assumption that a variant in a gene intolerant to functional genetic variation is more likely to be deleterious than one that is tolerant to functional variation. We used three intolerance scores based on NHLBI-ESP6500, ExAC datasets and a local dataset, all of which were developed with allele frequency data. The ExAC consortium has developed two additional scoring systems using large-scale exome sequencing data including intolerance scores (pLI) for loss-of-function variants and Z-scores for missense and synonymous variants. These were used for nonsense and missense variants respectively. In our final list, we also included missense variants in known tumor suppressor genes and oncogenes independent of their deleteriousness and intolerance scores. However, all variants had to meet previous cut-offs, i.e., MAF >0.1, pedigree segregation, CADD-PHRED >10 and positive conservation scores.

#### 2.5.3. Analysis of Non-Coding Variants

Non-coding regions make up over 98% of the genome and possess millions of potentially regulatory elements and noncoding RNA genes. Hence it is crucial to analyze the potential pathogenic impact of such variants in a Mendelian disease. Putative miRNA targets at variant positions within 3′ untranslated regions (UTRs) and 1 kb downstream of transcription end sites were detected by scanning the entire dataset of the human miRNA target atlas from TargetScan 7.0 [17] with the help of the intersect function of bedtools. We scanned the 5UTRs and 1 kb regions upstream of transcription start sites for transcription factor binding sites using SNPnexus (version 3; Dec 2017) [18]. For regulatory variants, we merged enhancer [19] and promoter [20,21] data from the FANTOM5 consortium and super-enhancer data from the super-enhancer archive (SEA) [22] and dbSUPER [23] using the intersect function of bedtools to identify putative enhancers, promoters and super-enhancers in our dataset. We accessed epigenomic data and marks from 127 cell lines from the NIH Roadmap Epigenomics Mapping Consortium via CADD v.1.3 [15], which gave us information on chromatin states from ChromHmm [24] and Segway [25]. The CADD analysis of 3′ UTRs also gave us mirSVR scores for putative miRNA targets; a score lower than -0.1 is indicative of a “good” miRNA target [26]. Furthermore, we used SNPnexus to obtain non-coding scores for each variant and to identify regulatory variants located in CpG islands. Top 3 ’UTR and downstream variants that had CADD scores >10 and miRNA target site matches with mirSVR scores <−0.1 were short-listed. Similarly, upstream and 5′ UTR variants in enhancers, promoters, super-enhancers or transcription factor binding sites with CADD scores >10 were selected.

### 2.6. Variant Validation

In order to increase the confidence in variant calls and reduce the risk of false positives, we visually inspected the sequencing data of all short-listed variants for correctness using the Integrative Genomics Viewer (IGV; version 2.4.10) [27].

### 2.7. Ingenuity Pathway Analysis (IPA)

IPA (Qiagen; http://www.qiagen.com/ingenuity; analysis date 08/04/2019) was used to perform a core analysis to identify relationships, mechanisms, functions, networks, and pathways relevant to the genes affected by variants that passed the mean allele frequency cut-off, fulfilled family-based segregation criteria, had CADD scores >10 and were not intergenic or intronic variants. Data were analyzed for all five families together. Top canonical pathways were identified from the IPA pathway library and ranked according to their significance to our input data. This significance was determined by p-values calculated using the right tailed Fisher’s exact test. These values indicated the probability of association of genes from the input dataset with the canonical pathway by random chance alone. Ratios were also calculated for each pathway by dividing the number of genes from the input dataset that map to the pathway by the total number of genes in that pathway. The ratios did not influence the ranking of the canonical pathways.

IPA was also used to generate gene networks in which upstream regulators were connected to the input dataset genes while taking advantage of paths that involved more than one link (i.e., through intermediate regulators). These connections represent experimentally observed cause-effect relationships that relate to expression, transcription, activation, molecular modification and transport as well as binding events.

### 2.8. STRING Analysis

A protein-protein interaction network was generated for each of the prioritized candidates using STRING (https://string-db.org; v11, 19/01/2019).

## 3. Results

### 3.1. Whole Genome Sequencing

In this study, five families with reported recurrence of NMTC were analyzed. WGS identified a total of 112254, 207873, 120323, 91427 and 101081 variants which were reduced by pedigree-based filtering to 6368, 9373, 3123, 7060 and 2708 in families 1-5, respectively. Non-synonymous SNVs were the most common exonic variants (Figure S1).

### 3.2. Final Prioritization of Candidates according to the FCVPPv2

After applying the FCVPPv2, the number of potential pathogenic protein coding variants was reduced to 31. These variants are listed in Table 1. A number of genes are of high significance to our study as they are either related to cancer or play a role in thyroid metabolism. *CHEK2* is a known tumor suppressor gene involved in DNA damage response [28]. *EWSR1* generates a powerful oncogenic protein causing Ewing sarcoma [29], *RET* is a proto-oncogene well-known in hereditary medullary thyroid carcinoma *NRP1* is known to be positively associated with the progression of breast cancer [30], *POT1* is a known predisposing gene in malignant melanoma [31] and *TG* encodes the precursor of iodinated thyroid hormones and is associated with susceptibility to autoimmune thyroid diseases (AITD) [32].

FCVPPv2 also identified 14 upstream and 5′ UTR variants, which are shown in Table 2. Among them, three variants are of particular interest in thyroid cancer. The *PCM1* variant is a 5′ UTR variant that our data showed to affect three transcription factor binding sites (Egr-3, AP-2alphaA and AP-2 gamma). Chromosomal aberrations involving this gene have been associated with PTC and a variety of hematological malignancies [33]. The other 5′ UTR variant is located in the *P4HB* gene which is known to be involved in the structural modification of the thyroglobulin precursor in hormone biogenesis [34]. Both variants are present in CpG islands and have been predicted to be localized at an active transcription start site by ChromHmm and Segway. The third variant is an upstream variant in the *DAPL1* gene, shown to affect the binding sites of MAZR and Sp1, a potential tumor suppressor in thyroid cancer, by SNPnexus and Segway.

Furthermore, 25 variants located downstream and in 3′ UTRs were shortlisted (Table 3). Among them, two genes of importance can be highlighted, namely *ACVR1B* and *NLK*. Mutations in the *ACVR1B* gene are associated with pancreatic cancer [35]. The variant in the 3′UTR of ACVR1B is localized at a target site for miR-6871-5p with a context ++ percentile score of 53, indicating a relatively good context for repression of the mRNA due to this miRNA. Altered expression of NLK is associated with cancer development and has been shown to be an independent prognostic factor in colorectal cancer [36]. The corresponding variant to this gene has two predicted miRNA target sites for miR-6818-5p and miR-6867-5p with high context ++ percentile scores (88 and 79, respectively).

Variants prioritized by the FCVPPv2 were also present in pathways, networks, and disease categories shown to be significantly enriched in FNMTC by IPA.

### 3.3. Ingenuity Pathway Analysis (IPA) Shows Enrichment of GPCR and RTK Mediated Pathways

In order to identify key biological functions and signaling pathways affected in FNMTC, we filtered the variants according to pedigree segregation, CADD scores and location, excluding intronic and intergenic variants. The variants were in 339 genes, with 92, 122, 14, 72 and 39 genes coming from families 1-5 respectively. Of these genes, 210 gene IDs could be mapped by IPA and were part of the subsequent analysis (Table S1). The remaining 129 genes were uncharacterized genes with RP11 IDs, and thus could not be mapped.

Of the top 150 diseases and bio functions, 123 were cancer-related with thyroid cancer at position 99 (p = 3.17 × 10^−5^), NMTC at position 120 (p = 6.39 × 10^−5^), differentiated thyroid cancer (DTC) at position 125 (p = 7.88 × 10^−5^) and PTC at position 148 (p = 2.16 × 10^−5^) (Table S1B). There was a high overlap of molecules among the four thyroid cancer related categories. This overlap of eight genes included two genes prioritized using our pipeline (*RET* and *TG*), that are of particular interest in thyroid cancer.

**Figure 3:**
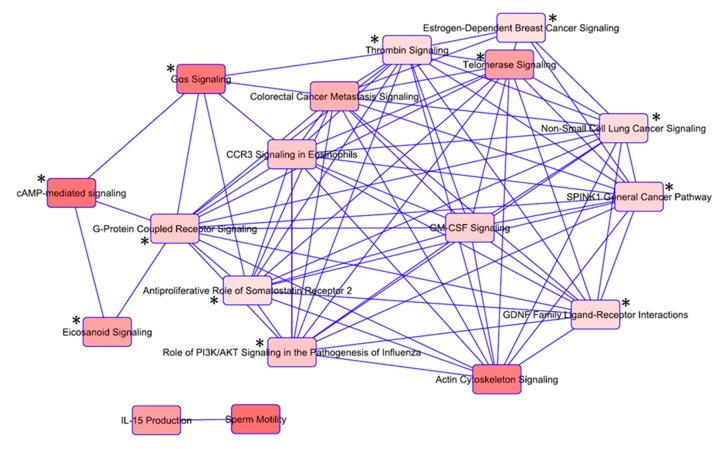
Top 18 overlapping canonical pathways visualized as a network, which shows each pathway as a single “node” colored proportionally to the Fisher’s Exact Test p-value, where brighter red indicates higher significance. Nodes marked with asterisk (*) belong to the group of GPCR and RTK mediated pathways.

With the aim of evaluating the canonical pathway results to determine the most significant pathways in our dataset, we created a network of the top 18 overlapping canonical pathways (Table S1C, Figure 3). The threshold of common genes between the pathways was set at 2. G-protein coupled receptor (GPCR) and receptor tyrosine kinase (RTK) mediated pathways, as major mediators of thyroid cancer development, were represented by 12 pathways (Figure 3). The genes involved in the top 18 pathways along with their corresponding variants are listed in Table S2.

### 3.4. Network Analysis Reinforces the Central Role of PI3K/AKT and MAPK/ERK Signaling in FNMTC

**Figure 4:**
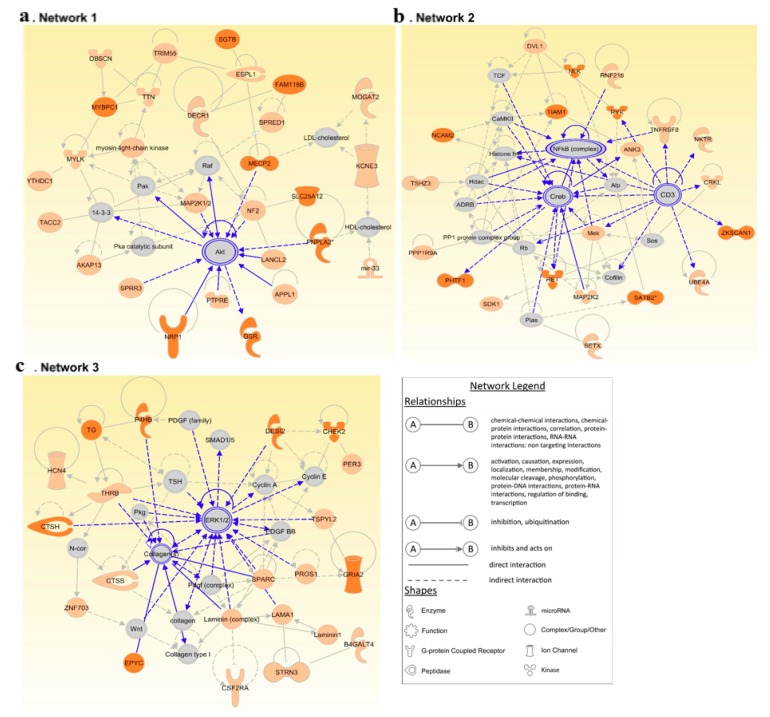
The top three molecular networks identified by Ingenuity Pathway Analysis (IPA): (**a**) Network 1. Protein Synthesis, Cardiovascular System Development and Function, Cellular Assembly and Organization; (**b**) Network 2. Cell Morphology, Cellular Assembly and Organization, Cellular Development and (**c**) Network 3. Endocrine System Disorders, Metabolic Disease, Organismal Injury and Abnormalities. Genes from our input-data that were prioritized based on pedigree segregation and PHRED-like CADD scores are shown in peach. Our top coding and non-coding candidates are highlighted in dark orange. Interactions of central genes of the network are highlighted in blue.

We conducted a network analysis using the IPA software to predict interacting molecular networks significant to our input-data and to evaluate genes with a central role in FNMTC (Figure 4, Table S1D). Since the IPA network analysis includes paths with intermediate regulators that involve more than one link, a comprehensive picture of the possible gene interactions was generated. The networks were ranked according to scores that were generated by considering the number of focus genes (input data) and the size of the network to approximate the relevance of the network to the original list of focus genes. We focused on the three highest scoring networks, which had scores ranging from 33 to 51 (Table S1D).

In coherence with the pathway analysis, the network analysis reinforces the importance of central perpetrators of GPCR and RTK mediated signaling (AKT, ERK1/2: Networks 1 & 3) and their downstream effectors (NFκB, CREB: Network 2). Furthermore, Network 3 encompasses a number of genes related to thyroid metabolism including *TG* from our prioritized shortlist.

### 3.5. Overlapping Pathways in Familial Non-Medullary Thyroid Cancer

**Figure 5:**
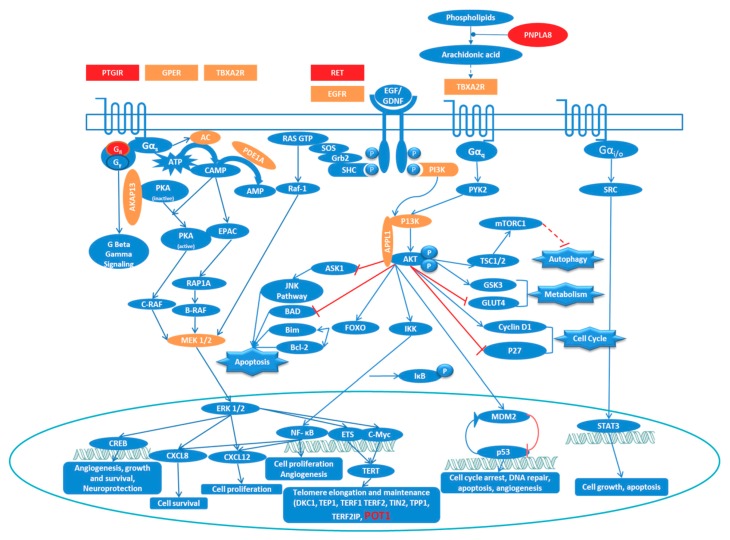
Proposed model for the most important molecular mechanisms in FNMTC. Genes from our input-data are highlighted in orange and genes corresponding to variants prioritized using the FCVPPv2 are highlighted in red.

Since GPCR and RTK mediated signaling were highlighted in both pathway and network analyses, we propose a pathway to facilitate a general understanding of FNMTC at a molecular level (Figure 5).

Activation of GPCR receptors can activate MAPK/ERK signaling as well as PI3K/AKT signaling via one of the four subclasses of G-proteins (G_αs_, G_αi/o_, G_αq/11_, and G_α12/13_). Dimerization of receptor-tyrosine kinase (RTK) receptors can be induced by growth factors such as EGFR and GDNF, which results in the phosphorylation and subsequent activation of the receptor monomers. Receptor activation is linked to downstream signal transduction pathways like the MAPK signaling cascade and the PI3K/AKT system via adaptor proteins. Genes from our dataset that were present in these pathways as activators or regulators are highlighted in Figure 5.

## 4. Discussion

The high heritability of thyroid cancer can be attributed to both rare, high-penetrance mutations and common, low-penetrance variants [4,13]. The former is best identified by studying families with a Mendelian pattern of inheritance of the disease in question. We used this principle in our study and identified 31 exonic and 39 non-coding rare potentially pathogenic variants segregating with the disease in five PTC-prone families.

Scientific and technological advancements in genomics have allowed WGS to become the state-of-the-art tool not only for the identification of driver mutations in tumors but also for the identification of novel cancer predisposing genes in Mendelian diseases. The former has led to improvements in personalized medicine, wherein therapeutic approaches are based on targeting dysregulated pathways specific to the affected individual. There are also some reports of WGS being successfully used to implicate rare, high-penetrance germline variants in cancer, for example *POT1* mutations in familial melanoma [39] and *POLE* and *POLD1* mutations in colorectal adenomas and carcinomas [40]. Identification of cancer-predisposing mutations is a critical step in cancer risk assessment and can help in cancer screening and prevention strategies. Furthermore, the implication of predisposition genes and their respective pathways may facilitate development of targeted therapy. However, one has to be critical in reporting novel variants before appropriate functional validation and evaluation of their penetrance in a large cohort of families. The importance of this step is exemplified by controversial findings regarding the implication of *HABP2 G534E* in familial NMTC [41].

Some of the genes shortlisted based on FCVPPv2 have already been identified in other cancers. These include *CHEK2* mutations in breast cancers and also in a variety of other cancers including thyroid cancer [28], *EWSR1* in Ewing sarcoma [29], *RET* in hereditary medullary thyroid carcinoma, *NRP1* in breast cancer [30] and germline *POT1* variants in malignant melanoma [31]. Moreover, it is interesting to note that the expression of *NRP2*, an important paralog of the *NRP1* gene, has been correlated to lymph node metastasis of human PTC and is required in the VEGF-C/NRP2 mediated invasion and migration of thyroid cancer cells [42]. The upstream variant in the *DAPL1* gene is shown to affect the binding sites of MAZR and Sp1 by SNPnexus and Segway. MAZR1, also known as PATZ1, has been shown to be downregulated and delocalized in thyroid cancer cell lines derived from papillary, follicular and anaplastic thyroid carcinomas [43]. Another study has demonstrated the role of PATZ1 as a tumor suppressor in thyroid follicular epithelial cells and its involvement in the dedifferentiation of thyroid cancer [44].

Other genes of interest shortlisted based on the pipeline (*PNPLA8*, *PTGIR*, *RET*, *GNB2* and *POT1*) were involved in the enrichment of MAPK/ERK and PI3K/AKT pathways. The MAPK pathway is the most frequently mutated signaling pathway in human cancer and is thus considered one of the most promising targets for cancer therapy. This pathway plays a central role in the induction of biological responses such as cell proliferation, differentiation, growth, migration and apoptosis [45]. Initiated by an extracellular mitogenic stimulus that leads to the activation of RTK or GPCR, the MAPK/ERK pathway leads to the phosphorylation and subsequent translocation of ERK into the nucleus. ERK activation plays a central role in the induction of cell cycle entry and the suppression of negative regulators of the cell cycle [46]. Although MEK1 and MEK2 can be activated by multiple MAP kinase kinase kinases (MAP3Ks) as well as by RAF, they serve as sole activators of ERK1/2 and thus as gatekeepers of the MAPK cascade [47]. Overexpression or aberrant activation of RTKs or their immediate downstream targets (PI3K, RAS and SRC) can result in the upregulation of the MAPK/ERK signaling pathway [48]. A common somatic mutation in this pathway is BRAFV600E, which has been implicated in melanoma [49], thyroid and colorectal cancer [50] and hairy cell leukemia [51].

The importance of the PI3K/AKT pathway in thyroid cancer was first recognized when patients suffering from Cowden’s syndrome caused by a germline mutation in the PTEN gene were found to have FTC [52]. PI3K activation phosphorylates and activates AKT which can have numerous downstream effects via activation or inhibition of multiple proteins that are involved in cell growth, proliferation, motility, adhesion, angiogenesis, metabolism and apoptosis.

Furthermore, our findings are in line with recent studies on PTC tissues and PTC cell lines have implicated activation of MAPK/ERK and PI3K/AKT pathways in thyroid carcinogenesis [53,54,55]. Interestingly, somatic alterations that lead to the activation of the MAPK pathway as well as of the PI3K/AKT pathway are common in aggressive thyroid cancers, such as metastatic or recurrent PTC/FTC and ATC [56]. The targeting of downstream RAS effectors has already been shown to be a promising approach, however patients treated with RAF or MEK inhibitors frequently develop drug resistance [47]. Targeting the downstream ERK kinase, which is also known as the gatekeeper of the MAPK cascade, can overcome the acquired drug resistance induced by upstream kinase inhibitors [57]. In this context, it is also important to note the similarity between our proposed model for the molecular mechanisms in FNMTC and the reported molecular mechanisms in non-familial NMTC. It is known that patients with familial NMTC may have a more aggressive form of the disease, with larger tumors in younger patients and increased rates of extra-thyroid extension and lymph node metastasis. This suggests that FNMTC should be explored further to gain a better understanding of the cause of increased aggressiveness. However, none of the variants were identified in more than one family. As the phenotypes of our families differed (as described in Figure 1), it is likely that also the mutations causing the disease in the families also are different. We analyzed only 5 families and no other WGS data on FNMTC are available, thus restricting the possibility to confirm the variants in larger data sets. Functional analysis of promising candidates highlighted in this study may shed some light to the mechanisms underlying this phenomenon.

Interpreting WGS data and selecting one out of millions of genetic variants as the cause of hereditary cancer is a daunting task and highlights the importance of the use of a standardized protocol like the FCVPPv2. We were able to prioritize 31 exonic and 39 non-coding potential cancer-predisposing variants using our family-based pipeline from which we hope to pinpoint one candidate gene for each family. The final selection and implication of one candidate gene predisposing to cancer in each family is beyond the scope of this paper as it will involve further steps including population screening and functional studies. In the present study, we decided to focus on the analysis of pathways that are enriched in familial NMTC to see how the variants prioritized using our pipeline fit into the general pathway analysis results. The IPA analysis of all genes already presented us with valuable data and there was a high involvement of genes prioritized using our pipeline in the top diseases and bio functions, canonical pathways and networks generated by IPA. Although IPA could give us a general idea of molecular pathways affected in the studied families, it is important to keep in mind that the analysis was conducted at a gene level and not at a variant level. The evaluation at a variant level is largely dependent on the pipeline and its subsequent steps as mentioned above. We have already successfully implemented this pipeline to identify *DICER1* as a candidate predisposing gene in familial Hodgkin lymphoma [58] and are confident that our pipeline can be applied to the NMTC families in a similar manner.

## 5. Conclusions

In conclusion, WGS data analysis of five NMTC-prone families allowed us to prioritize 31 exonic and 39 non-coding variants from which we subsequently hope to identify one candidate gene per family. Furthermore, we were able to identify pathways and networks significant to our dataset, including important tumorigenic pathways such as MAPK/ERK and PI3K/AKT signaling pathways. The implication of previously reported tumorigenic signaling pathways and the presence of known tumor suppressor or oncogenes in these affected pathways show that the pathogenesis of FNMTC is in concordance with characteristic molecular mechanisms of cancer. The next steps will include selecting one candidate gene per family and validating it with the help of population screening and functional studies. We hope that our results can facilitate personalized therapy in the studied families and contribute to the screening of other individuals at risk of developing NMTC.

## Figures and Tables

**Table 1:** Top exonic variants prioritized following the FCVPPv2. Chromosomal positions, classifications, PHRED-like CADD scores and the percentage of positive intolerance (Int) and deleteriousness (Del) scores are included for each variant. Additional information regarding protein-protein interactions (STRING), localization in protein domains (InterPro [37]) and the biological function of the respective protein (GeneCards [38]) is included.

Family	Gene	Chrom_Pos_Ref_Alt	Exonic Classification	CADD_PHRED Score	Int (%)	Del (%)	Interactions (STRING)	Domain	Function
**1**	CHEK2	22_29107974_C_T	nonsynonymous SNV	24.8	75	42	ATM, ATR, CDC25C, CDC25A, TP53BP1, TP53, MRE11A, BRCA1, RAD50, H2AFX	Serine/ threonine-protein kinase-like domain	DNA repair, cell cycle arrest or apoptosis in response to DNA damage; **tumor suppressor gene**
**1**	SLC35A4	5_139947647_T_C	nonsynonymous SNV	26.5	50	75	SCAMP3, PRKAA1, SLC35B2, SLC35D2, ABCB10	Nucleotide-sugar transporter	Pyrimidine nucleotide-sugar transmembrane transporter activity, sialic acid transmembrane transporter activity
**1**	ANXA3	4_79531211_C_T	nonsynonymous SNV	27.9	50	75	STX4, SNAP23, STXBP2, ANXA11, ANXA4, FPR1, CACNA1B, NLRP3, SUMF1, FPR2	Annexin repeat, conserved site	Phospoholipase A2 inhibition, anti-coagulant properties, formation of inositol 1-phosphate from inositol 1,2-cyclic phosphate
**1**	EWSR1	22_29687556_C_A	nonsynonymous SNV	22.7	100	75	BARD1, ETV1, TAF5, TAF5L, FUS, TAF12, DHX9, TP53, PIOK2, POLR2G	NA	Gene expression, cell signaling, RNA processing and transport; **oncogene**
**1**	RTTN	18_67776873_G_A	nonsynonymous SNV	26.7	25	83	INVS, LEFTY2, DNAH11, CCDC102B, EN1, CCDC178, L3MBTL4, CHML, CHM, DLL1	NA	Involved in the genetic cascade that governs left-right specification and in the maintenance of a normal ciliary structure.
**1**	TIAM1	21_32526579_G_A	nonsynonymous SNV	35	100	92	CDC42, SRC, RAC1, EFNB1, RAC2, NME1, EPHA2, RHOA, PARD6A, ARF6	Dbl homology (DH) domain	Modulates the activity of Rho GTP-binding proteins, connects extracellular signals to cytoskeletal activities, activates Rac1, CDC42, and to a lesser extent RhoA.
**1**	MAN2B2	4_6612617_C_T	nonsynonymous SNV	34	25	100	MAN2C1, NAAA, SIAE, GLB1L3, GLB1, PYGB, PYGL, PYGM, NAGA	Glycosyl hydrolase family 38, C-terminal	carbohydrate binding, alpha-mannosidase activity, involved in metabolism and other glycan degradation
**2**	CLEC18B	16_74446758_G_A	nonsynonymous SNV	23.3	50	67	FRAS1, LEO1, FREM2	Epidermal growth factor-like domain	Ca^2+^ independent binding of polysaccharides
**2**	PTGIR	19_47124811_C_T	nonsynonymous SNV	35	100	67	HTR7, NPS, AVP, VIP, ADM, AVPR2, ADRB2, PTH, ADCY6, GNB1	NA	Member of GPCR family 1, receptor for prostacyclin, elicits potent vasodilation and inhibition of platelet aggregation
**2**	UBN1	16_4911084_G_A	nonsynonymous SNV	34	75	67	ASF1A, HIRA, CABIN1, RB1, TP53, EP400, HMGA1, HMGA2, H1F0, HIST1H1C	Ubinuclein middle domain	Novel regulator of senescence, involved in DNA damage/telomere stress induced senescence and cellular senescence, required for replication independent chromatin assembly
**2**	GALNT10	5_153789322_G_C	nonsynonymous SNV	24.6	100	67	MUC7, MUC1, C1GALT1, MUC5AC, GCNT1, ST6GALNAC1, B3GNT6, MUC2, MUC16, C1GALT1C1	Ricin B-related lectin	Catalyzes the initial reaction in O-linked oligosaccharide biosynthesis
**2**	OSGIN2	8_90921899_A_T	nonsynonymous SNV	23.7	100	67	CALB1, CA7, DECR1, DECR2, CALB2, NBN, SLC39A3	NA	Possibly involved in meiosis or the maturation of germ cells, associated with retinitis pigmentosa
**2**	TG	8_133900661_A_C	nonsynonymous SNV	25	0	75	TPO, LRP2, TSHR, ASGR1, NKX2-1, INS, SLC5A5, PAX8, ASGR2, ALB	Thyroglobulin type-1 domain	Precursors of iodinated **thyroid hormones** (T4) and triiodothyronine (T3), associated with susceptibility to **autoimmune thyroid diseases** (AITD)
**2**	GSR	8_30585111_C_T	nonsynonymous SNV	34	100	75	GPX1, GPX3, GPX2, CAT, GPX4, GSS, GPX7, HPGDS, TXN, ACLY	Pyridine nucleotide-disulphide oxidoreductase, FAD/NAD(P)-binding domain	Oxidoreductase activity and flavin adenine dinucleotide binding
**2**	KCNT1	9_138676399_A_G	nonsynonymous SNV	11.1	100	75	GPR55, C11orf40, ASRGL1, SLC11A1	NA	Sodium/Chloride/Calcium-activated potassium channel subunit, activated upon stimulation of GPCRs
**2**	KLHL18	3_47385160_A_G	nonsynonymous SNV	27.4	100	75	COPS5, GPKOW, CNIH4, COPS6, PDE7A, CNIH3, PDE/B, PDE6D, EEF1G, CNIH2	Galactose oxidase, beta-propeller	Involved in the ubiquination process, specific role has yet to be elucidated
**2**	CDRT1, RP11-385D13.1	17_15501921_G_A	nonsynonymous SNV	25.3	-	83	-	WD40/YVTN repeat-like-containing domain	CDRT1: a protein-ubiquitin ligase; RP11: a component of the spliceosome complex, one of several retinitis pigmentosa-causing genes
**2**	RET	10_43600559_T_C	nonsynonymous SNV	26.3	75	83	GDNF, GFRA1, NRTN, SHC1, PSPN, PIK3CA, GFRA2, PIK3CD, PIK3CB, GRB2	Cadherin-like domain	**Proto-oncogene**, receptor tyrosine kinase; involved in cell differentiation, growth, migration and survival
**2**	SCN10A	3_38755465_C_A	nonsynonymous SNV	35	50	92	SCN5A, CALM2, SCN8A, SCN2A, SCN11A, SCH3A, SCN1A, SCN9A, SCN4A, SCN1B	Ion transport	Tetrodotoxin-resistant channel that mediates the voltage-dependent sodium ion permeability of excitable membranes, plays a role in neuropathic pain mechanisms
**3**	C1orf27	1_186355211_G_A	nonsynonymous SNV	25.1	0	67	DRAM1, PID1, TXLNG	ODR-4-like domain	Possible involvement in the trafficking of a subset of GPCRs
**3**	CPXM1	20_2776248_C_T	nonsynonymous SNV	32	100	75	FAM196A, PPP2R2B, SEC13	Peptidase M14, carboxypeptidase A	Binds collagen, involved in adipogenesis through extracellular matrix remodeling, may act as a TSG in breast cancer
**3**	ZBTB41	1_197128680_C_T	nonsynonymous SNV	23.1	100	75	POTEE, POTEI, POTEJ, POTEF, SKIV2L, CFHR4, RIPK4, PHLPP2, PHLPP1, C7orf73	NA	May be involved in transcriptional regulation
**3**	AR	X_66765158_T_TGCAGCAGCA	nonframeshift insertion	12.8	67	-	NCOA2, NCOA4, KLK3, KDM1A, FOXA1, SRC, HSP90AA1, FKBP5, NCOA1, CCND1	Androgen receptor domain	Steroid-hormone activated transcription factor. Stimulates transcription of androgen responsive genes.
**4**	PKHD1L1	8_110477162_G_A	nonsynonymous SNV	27.5	0	100	TMEM2, CUEDC1, PKHD1, PKD1P1, C2orf74, RAD21-AS1, FAM135B, CSMD3, MUM1L1, HSPA12B	NA	Signaling receptor activity, immune response
**4**	ECE2	3_184008594_G_C	nonsynonymous SNV	32	75	100	RPS6KA2, EDN3, EDNRA, DHX40, MYSM1. EDNRB, EDN1, EZR, LARP6, PRKCE	Peptidase M13, neprilysin, C-terminal/Metallopeptidase, catalytic domain	Metalloprotease involved in the generation of functionally pleiotropic members of the endothelin vasoactive family, possibly involved in amyloid-beta processing
**5**	EPYC	12_91365726_C_G	nonsynonymous SNV	27	25	67	RIPK4, PPIE, POTEI, POTEE, POTEJ, POTEF, PRKAR1B, PRKAR1A, CNBD2, PRKAR2B	Leucine-rich repeat	Regulates fibrillogenesis by interacting with collagen fibrils and other extracellular matrix proteins
**5**	SPOCK1	5_136448179_G_A	nonsynonymous SNV	25.7	100	67	SPARC, MMP16, FST, MMP14, SPARCL1, MMP2, CITED2, CHD1L, CFTR, HMCN1	Proteinase inhibitor I1, Kazal	Calcium ion binding, cysteine-type endopeptidase inhibitor activity, cell-cell interactions, may contribute to various neuronal mechanisms
**5**	MYBPC1	12_102046527_A_G	nonsynonymous SNV	25.9	100	67	MYH3, TTN, TNNT3, NEB, TNNI2, DMD, MYL1, TMOD4, TNNI1, MYL3	Immunoglobulin subtype	Member of the myosin-binding protein C family, binds actin and titin, modulates muscle contraction
**5**	ACSS3	12_81593172_T_G	nonsynonymous SNV	32	100	83	ALDH2, ALDH3A2, EHHADH, ACLY, ECHDC1, ACADM, ALDH6A1, ALDH9A1, ALDH1B1	AMP-dependent synthetase/ligase	Activates acetate for use in lipid synthesis or energy generation
**5**	NRP1	10_33469205_G_C	nonsynonymous SNV	24.2	75	83	SEMA3A, KDR, FLT1, PLXNA1, PLXNA2, SEMA3C, PLXNA4, SEMA3F, PLXNA3, SEMA3E	Neuropilin-1, C-terminal	Membrane-bound coreceptor to a tyrosine kinase receptor for both VEGF and semaphorin family members; plays roles in angiogenesis, axon guidance, cell survival, migration and invasion
**5**	POT1	7_124532359_C_A	nonsynonymous SNV	32	50	92	TERF1, TINF2, ACD, TERF2, TERF2IP, RAD50, MRE11A, H2AFx, DCLRE1B, BRCA1	Nucleic acid-binding, OB-fold	Member of the shelterin complex; involved in regulating telomere length and protecting chromosome ends from illegitimate recombination, catastrophic instability and abnormal segregation

**Table 2:** Top upstream and 5′ UTR variants prioritized according to the FCVPPv2. Variant annotation, chromosomal position, and regulatory consequences according to FANTOM5, SEA, CADD and SNPnexus are listed. The FANTOM5 database gives information on known promoters. CADD gives an overall deleteriousness score together with chromatin state information based on ChromHmm and Segway scores and information on transcription factor binding sites (TFBSs). Location of the variants within a specific TFBS and CpG island were obtained from SNPnexus. A cumulative non-coding score is shown as a percentage of positive scores from all scores listed in the footnote. Cut-offs for these scores are also indicated in the footnote.

Variant Details				FANTOM5, SEA	Annotations From CADD						SNPnexus		
**F^I^**	Gene	Variation_ Annotation	Chrom_Pos_Ref_Alt	Promoter/Enhancer_ Start..End, Strand	CADD_PHRED Score	Chromatin State^II^			TFBS		TFs	In a CpG Island?	Non-coding scores (%)^III^
						Chrom-Hmm State	Score	Segway	TFBS	TFBS Peaks^I^			
**1**	PCM1	SNV_UTR5	8_17780410_G_A	−	17.2	TssA	0.95	TSS	50	92	Egr-3, AP-2alphaA, AP-2gamma	Yes	67
**1**	STAP1	SNV_UTR5	4_68424468_A_G	Promoter_68424462..68424469,+	15.4	Quies	0.71	GM0	18	24	−	No	71
**1**	DAPL1	SNV_ Upstream	2_159651789_C_T	−	13.1	−	−	TF0	1	2	MAZR, Sp1	No	50
**2**	LRRC48	SNV_UTR5	17_17876279_G_T	−	10.8	TssA	0.803	GS	28	56	−	No	50
**2**	P4HB	SNV_UTR5	17_79818442_T_G	−	11.4	TssA	0.945	TSS	51	78	−	Yes	60
**2**	FAM118B	SNV_UTR5	11_126081608_C_T	−	10.5	TssA	0.969	TSS	60	129	−	Yes	33
**2**	AZIN1	SNV_UTR5	8_103876327_G_A	−	12.4	TssA	0.929	TSS	47	76	−	No	50
**2**	RPS3A	SNV_UTR5	4_152020778_C_G	Promoter_152020736..152020788,+	16.0	TssA	0.961	TSS	81	184	−	Yes	86
**3**	C20orf194	SNV_ Upstream	20_3388577_C_A	−	13.4	TssA	0.921	TSS	17	26	Egr-2, Egr-3	Yes	50
**4**	DNAI1	SNV_UTR5	9_34458888_T_C	Promoter_34458851..34458908,+	14.4	TssAFlnk	0.575	GM1	10	13	−	Yes	71
**4**	PNPLA2	SNV_UTR5	11_819602_G_C	Promoter_819601..819612,+	10.6	TssA	0.945	TSS	38	65	−	Yes	50
**4**	GNB2	Indel_UTR5	7_100271438_G_GCGCCGCCGCCGC	−	17.5	TssA	0.992	TSS	65	115	CUTL-1	Yes	25
**4**	PHTF1	SNV_UTR5	1_114301745_G_T	−	16.2	TssA	0.961	TSS	20	28	CREB, delta CREB	Yes	50
**4**	ZKSCAN1	SNV_UTR5	7_99613211_C_G	−	21.4	TssA	0.937	TSS	65	140	Elk-1, LCR-F1	Yes	67

**[I]** = Family ID, **[II]** = ChromHmm and Segway; ChromHmm shows the proportion of 127 cell types in a particular chromatin state (x). Scores closer to 1 indicate a higher proportion of cell types in the specified chromatin state. X can be the following: active transcription start sites (TssA), enhancers (Enh), bivalent TSS (TssBiv), bivalent enhancers (EnhBiv), genic enhancers (EnhG), flanking transcription states (TxFlnk), flanking bivalent TSS (TssBiv), active transcription flanking sites (TssAFlnk), transcription states (Tx) and weak transcription states (TxWk), repressed polycomb (ReprPC) and weak repressed polycomb regions (PeprPCWk), heterochromatin (Het) and quiescent regions (Quies). Segway is a software that transforms multiple datasets on chromatin properties into a single annotation of the genome. The annotations can be as follows: D: dead, F: FAIRE, R: repression, H3K9me1: histone 3 lysine 9 monomethylation, L: low, GE: gene end, TF: transcription factors, C: CTCF, TSS: transcription start site, GS: gene start, E: enhancer, GM: gene middle and ZnfRpts: zinc finger repeats. **[III]** = Non-coding scores with their cut-offs in brackets: FitCons Score (≥0.2), FitCons P-Value (≤0.05), EIGEN (>0, at least 1 of 2 must be positive), FatHMM (>0.5), GWAVA (>0.4, at least 2 of 3 must be positive), DeepSEA (>0.5, at least 2 of 3 must be positive), FunSeq2 (>3), ReMM (>0.5). **[IV]** = TFBS peaks: regions with enrichment of transcription factor binding sites (TFBS).

**Table 3:** Top downstream and 3′ UTR variants prioritized according to the FCVPPv2. Variant annotation, chromosomal position, and regulatory consequences according to TargetScan, CADD and SNPnexus are listed. Information on miRNA target sites from TargetScan and chromatin states from CADD are also included. A cumulative non-coding score is shown as a percentage of positive scores from all scores listed in the footnote. Cut-offs for these scores are also indicated in the footnote.

Variant Details				TargetScan			Annotations from CADD					SNP-nexus
F^I^	Gene	Variation_ Annotation	Chrom_Pos_Ref_Alt	miRNA Target Sites	Context Score ++ Percentile^II^	Site Type	mirSVR-Score	CADD_ PHRED Score	Chromatin State^III^			Non-Coding Scores ^IV^ (%)
									Chrom-Hmm	Score	Segway	
**1**	DESI2	SNV_UTR3	1_244872281_A_G	DESI2:miR-3651	94	7mer-m8	-0.84	15.6	TxWk	0.73	R2	60
**1**	DPYSL3	SNV_UTR3	5_146770537_A_T	DPYSL3:miR-4693-5p, DPYSL3:miR-4768-3p, DPYSL3:miR-6888-5p	20, 52, 59	7mer-1a, 7mer-m8, 7mer-m8	-0.24	11.1	−	−	L1	40
**1**	MECP2	SNV_UTR3	X_153295452_G_A	MECP2:miR-6812-3p	72	7mer-1a	NA	10.4	Tx	0.46	TF2	25
**1**	RYK	SNV_UTR3	3_133876591_C_T	RYK:miR-548aq-3p/548am-3p/548aj-3p/548ah-3p/548ae-3p/548j-3p/548x-3p; RYK:miR-5582-3p	93, 95	7mer-m8, 7mer-m8	−1.25	12.7	TxWk	0.50	F1	80
**1**	SGTB	SNV_UTR3	5_64965337_A_C	SGTB:miR-3187-3p, SGTB:miR-4529-5p	84, 46	7mer-m8, 7mer-1a	−0.75	16.8	TxWk	0.68	GE0	67
**1**	SLC25A12	SNV_UTR3	2_172641178_G_A	SLC25A12:miR-3622b-5p	62	7mer-1a	−0.31	15.1	−	−	GE1	60
**2**	ACVR1B	SNV_UTR3	12_52388057_A_G	ACVR1B:miR-6871-5p	53	7mer-m8		14.8	−	−	−	60
**2**	NCAM2	Indel_UTR3	21_22913891_AT_A	NCAM2:miR-6885-3p	46	7mer-m8	NA	11.3	Quies	0.99	F0	50
**2**	NOP2	SNV_UTR3	12_6666047_A_T	NOP2:miR-3662	98	7mer-1a	−1.29	14.2	Tx	0.48	GE0	50
**2**	NUPL1	SNV_UTR3	13_25909315_T_C	NUPL1:miR-3145-3p	69	8mer	−	11.3	−	−	−	80
**2**	PNPLA8	SNV_UTR3	7_108112453_A_G	PNPLA8:miR-3163, PNPLA8:miR-4668-3p, PNPLA8:miR-551b-5p	65, 56, 62	7mer-m8, 7mer-1, 7mer-m8	−1.25	13.3	TxWk	0.53	F0	80
**2**	STK32A	SNV_UTR3	5_146763869_G_A	STK32A:miR-4484, STK32A:miR-548an, STK32A:miR-6768-3p	99, 80, 74	8mer, 7mer-1a, 7mer-1a	NA	11.8	Quies	0.48	F1	40
**2**	SVEP1	SNV_UTR3	9_113128472_T_C	SVEP1:miR-1468-3p	96	7mer-m8	−1.32	17.0	Quies	0.77	F1	60
**2**	TFCP2	Indel_UTR3	12_51487616_A_AACAC	TFCP2:miR-8485	95	7mer-m8	NA	10.2	Tx, TxWk	0.47, 0.52	GE0	67
**2**	MRPL51	SNV_ downstream	12_6600160_C_T	MRPL51: miR-6802-3p	90	7mer-m8	NA	13.4	TxWk	0.63	H3K9 me1	50
**2**	ZNF45	SNV_ncRNA_UTR3	19_44417402_A_G	ZNF45: miR-6777-3p	96	8mer	−0.39	11.3	ZnfRpts	0.78	GE1	60
**3**	NLK	Indel_UTR3	17_26522009_T_TCACA	NLK:miR-6818-5p, NLK:miR-6867-5p	88, 79	7mer-m8, 8mer	−0.62	11.7	TxWk	0.63	TF1	100
**4**	ADAMTS1	SNV_UTR3	21_28208629_T_C	ADAMTS1:miR-325, ADAMTS1:miR-628-3p	88, 97	7mer-1a, 8mer	−1.31	16.0	TxWk	0.58	F1	60
**4**	GRIA2	SNV_UTR3	4_158284635_G_A	GRIA2:miR-486-5p, GRIA2:miR-7152-5p	88, 84	7mer-1a, 7mer-m8	−0.87	22.3	Quies	0.84	L1	60
**4**	IGSF9	Indel_UTR3	1_159896866_TCACA_T	IGSF9:miR-377-3p, IGSF9:miR-5582-3p, IGSF9:miR-8485	98, 82, -1	8mer,7mer-m8, 3’compensatory	−0.92	17.0	−	-	TF1	50
**4**	MPP6	SNV_UTR3	7_24727611_A_G	MPP6:miR-138-2-3p, MPP6:miR-205-3p, MPP6:miR-498	93, 50, 48	7mer-m8, 7mer-1a, 7mer-1a	NA	15.5	TxWk, Quies	0.50, 0.45	GE0	60
**4**	ZNF532	SNV_UTR3	18_56651809_T_C	ZNF532: miR-1277-5p	53	7mer-m8	−0.86	15.2	TxWk	0.73	R0	80
**5**	KLF7	Indel_UTR3	2_207945783_ATATGTG_A	KLF7:miR-511-3p, KLF7:miR-223-5p	82, 59	7mer-1a, 7mer-1a	−1.10	11.9	Tx	0.73	F1	50
**5**	SATB2	SNV_UTR3	2_200134548_A_G	SATB2:miR-3156-5p, SATB2:miR-3126-3p, SATB2:miR-4720-5p/4799-3p/5588-5p, SATB2:miR-3128, SATB2:miR-6868-5p	37, 86, 74, 76, 83	7mer-m8, 7mer-m8, 7mer-1a, 8mer, 7mer-1a	-1.22	15.2	Quies	0.74	F1	60
**5**	ZNF608	SNV_ downstream	5_123972606_C_A	ZNF608: miR-4786-3p	87	7mer-m8	NA	16.8	TxWk	0.69	D	60

**[I]** = Family ID, **[II]** = Context score ++ percentile: a higher percentile score indicates a better context for repression of an mRNA due to a miRNA, **[III]** = ChromHmm and Segway; ChromHmm shows the proportion of 127 cell types in a particular chromatin state (x). Scores closer to 1 indicate a higher proportion of cell types in the specified chromatin state. X can be the following: active transcription start sites (TssA), enhancers (Enh), bivalent TSS (TssBiv), bivalent enhancers (EnhBiv), genic enhancers (EnhG), flanking transcription states (TxFlnk), flanking bivalent TSS (TssBiv), active transcription flanking sites (TssAFlnk), transcription states (Tx) and weak transcription states (TxWk), repressed polycomb (ReprPC) and weak repressed polycomb regions (PeprPCWk), heterochromatin (Het) and quiescent regions (Quies). Segway is a software that transforms multiple datasets on chromatin properties into a single annotation of the genome. The annotations can be as follows: D: dead, F: FAIRE, R: repression, H3K9me1: histone 3 lysine 9 monomethylation, L: low, GE: gene end, TF: transcription factors, C: CTCF, TSS: transcription start site, GS: gene start, E: enhancer, GM: gene middle and ZnfRpts: zinc finger repeats. **[IV]** = Non-coding scores with their cut-offs in brackets: FitCons Score (≥ 0.2), FitCons P-Value (≤0.05), EIGEN (> 0, at least 1 of 2 must be positive), FatHMM (>0.5), GWAVA (>0.4, at least 2 of 3 must be positive), DeepSEA (>0.5, at least 2 of 3 must be positive), FunSeq2 (>3), ReMM (>0.5).

## Supplementary Materials

The following are available online at https://www.mdpi.com/2218-273X/9/10/605/s1, Figure S1: Variant Distribution, Table S1: Ingenuity Pathway Analysis Results, Table S2: Gene List from Top 20 Canonical Pathways.
