# Supplementary material for: Whole Genome Sequencing of Familial Non-Medullary Thyroid Cancer Identifies Germline Alterations in MAPK/ERK and PI3K/AKT Signaling Pathways
Source: Biomolecules. 2019 Oct 13;9(10):605. doi: 10.3390/biom9100605 (PMC6843654; doi:10.3390/biom9100605)
Supplement: Supplementary file 1 [file biomolecules-09-00605-s001.zip › Figure S1 Variant Distribution.pdf]

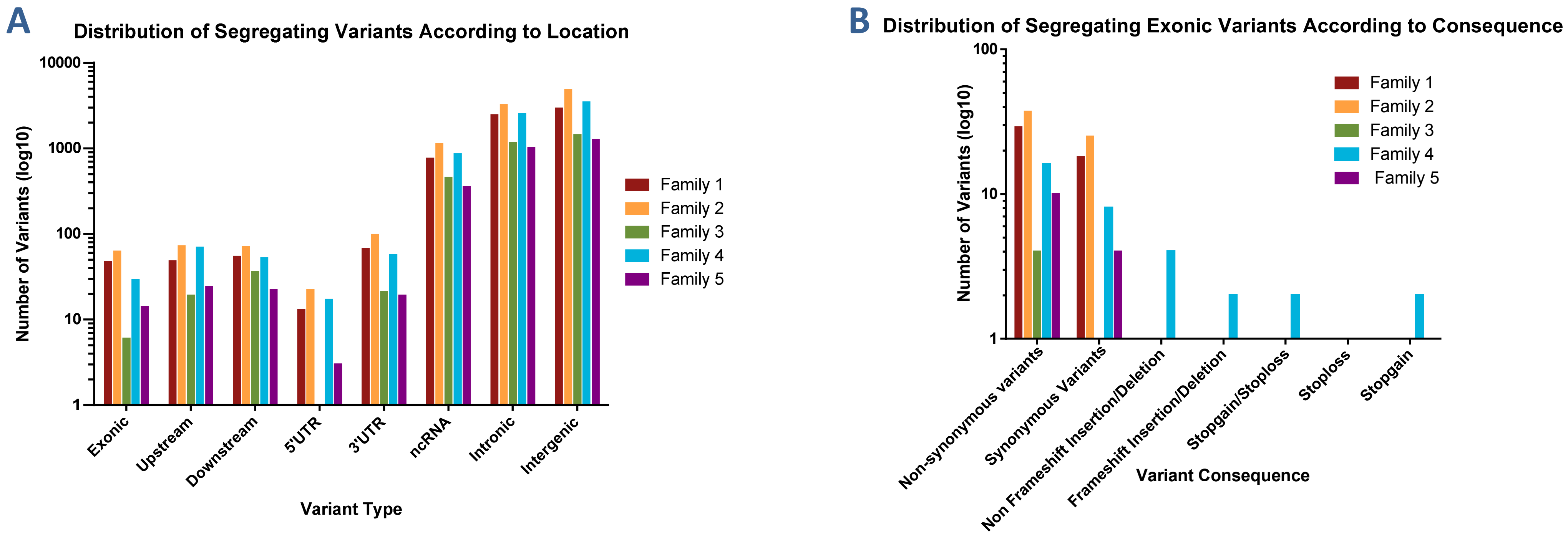


**Figure S1.** (a) Location-based distribution of all variants segregating in each family; (b) Distribution of exonic variants segregating in each family according to their consequence on the protein sequence.
