# Supplementary material for: Whole Genome Sequencing of Familial Non-Medullary Thyroid Cancer Identifies Germline Alterations in MAPK/ERK and PI3K/AKT Signaling Pathways
Source: Biomolecules. 2019 Oct 13;9(10):605. doi: 10.3390/biom9100605 (PMC6843654; doi:10.3390/biom9100605)
Supplement: Supplementary file 1 [file biomolecules-09-00605-s001.zip › Table S2 Gene List from Top 20 Canonical Pathways.pdf]

Table S2. List of genes present in the top 20 canonical pathways predicted by IPA. The position, annotation and PHRED-like CADD scores corresponding to the variants of these genes have been listed in this table. Genes corresponding to variants shortlisted using FCVPPv2 are marked in bold.

Family	Gene ID	Chr_Pos_Ref_Alt	Annotation	CADD_Phred	Present in the following top canonical pathways
1	HSD17B4	5_118878833_G_A	downstream	12.5	Estrogen-dependent breast cancer signaling
1	MAP2K2	19_4124798_T_A	Upstream	13.6	cAMP-mediated signaling, Gαs signaling, actin cytoskeleton signaling, telomerase signaling, colorectal cancer metastasis signaling, role of PI3K/AKT signaling in the pathogenesis of influenza, CCR3 signaling in eosinophils, G-protein coupled receptor signaling, GM-CSF signaling, SPINK1 general cancer pathway, GDNF family ligand-receptor interactions, thrombin signaling, non-small cell lung cancer signaling, anti-proliferative role of somatostatin receptor 2
1	MYLK	3_123331882_A_T	ncRNA_UTR3	12.8	Actin cytoskeleton signaling, CCR3 signaling in eosinophils, thrombin signaling
1	PDE1A	2_183005299_G_C	UTR3	11.7	Sperm motility, cAMP-mediated signaling, G-protein coupled receptor signaling
1	**RYK**	3_133876591_C_T	UTR3	12.7	Sperm motility, IL-15 production
1	TBXA2R	19_3594840_C_G	nonsynonymous SNV	19.4	cAMP-mediated signaling, eicosanoid signaling, G-protein coupled receptor signaling
1	**TIAM1**	21_32526579_G_A	nonsynonymous SNV	35	Actin cytoskeleton signaling
1	TTN	2_179422237_C_T	nonsynonymous SNV	23.1	Actin cytoskeleton signaling
2	APPL1	3_57261616_G_A	upstream	11.6	Colorectal cancer metastasis signaling
2	CRKL	22_21270749_C_A	upstream	11.4	Actin cytoskeleton signaling, Role of PI3K/AKT signaling in the pathogenesis of influenza
2	CSF2RA	X_1422861_T_C	stoploss SNV	14.5	Sperm motility, IL-15 production, GM-CSF signaling
2	DVL1	1_1275695_G_A	nonsynonymous SNV	24	Colorectal cancer metastasis signaling
2	EGFR	7_55259429_G_A	synonymous SNV	11.1	Sperm motility, IL-15 production, telomerase signaling, colorectal cancer metastasis signaling, SPINK1 general cancer pathway, thrombin signaling, non-small cell lung cancer signaling, estrogen-dependent breast cancer signaling
2	**GSR**	8_30585111_C_T	nonsynonymous SNV	34	Glutathione redox reactions II
2	PIK3R5	17_8790439_G_A	nonsynonymous SNV	22.3	Actin cytoskeleton signaling, telomerase signaling, colorectal cancer metastasis signaling, role of PI3K/AKT signaling in the pathogenesis of influenza, CCR3 signaling in eosinophils, G-protein coupled receptor signaling, GM-CSF signaling, SPINK1 general cancer pathway, GDNF family ligand-receptor interactions, thrombin signaling, non-small cell lung cancer signaling, anti-proliferative role of somatostatin receptor 2, estrogen-dependent breast cancer signaling
2	**PNPLA8**	7_108112453_A_G	UTR3	13.3	Sperm motility, eicosanoid signaling
2	**PTGIR**	19_47124811_C_T	nonsynonymous SNV	35	cAMP-mediated signaling, Gαs signaling, eicosanoid signaling, G-protein coupled receptor signaling

**2**	**RET**	10_43600559_T_C	nonsynonymous SNV	26.3	Sperm motility, IL-15 production, GDNF family ligand-receptor interactions
**4**	AKAP13	15_86287866_C_T	synonymous SNV	12.8	cAMP-mediated signaling
**4**	**GNB2**	7_100271438_G_ GCGCCGCCGCCGC	UTR5, Indel	17.2	Gαs signaling, colorectal cancer metastasis signaling, CCR3 signaling in eosinophils, thrombin signaling, anti-proliferative role of somatostatin receptors
**4**	GPER1	7_1131887_C_T	nonsynonymous SNV	22	cAMP-mediated signaling, Gαs signaling, G-protein coupled receptor signaling
**5**	**ACSS3**	12_81593172_T_G	nonsynonymous SNV	32	Acetate conversion to Acetyl-CoA
**5**	**POT1**	7_124532359_C_A	nonsynonymous SNV	32	Telomerase signaling
